# Expanding Robotic Arm-Assisted Knee Surgery: The First Attempt to Use the System for Knee Revision Arthroplasty

**DOI:** 10.1155/2020/4806987

**Published:** 2020-02-12

**Authors:** Dimitrios Kalavrytinos, Christos Koutserimpas, Ioannis Kalavrytinos, Konstantinos Dretakis

**Affiliations:** ^1^Department of Health Sciences, University of Patras, Greece; ^2^2nd Department of Orthopaedics, “Hygeia” General Hospital of Athens, Greece; ^3^Department of Orthopaedics and Traumatology, “251” Hellenic Air Force General Hospital of Athens, Greece; ^4^4th Department of Orthopaedics, “Hygeia” General Hospital of Athens, Greece

## Abstract

Robotic arm-assisted arthroplasty was introduced in 2006 and has expanded its applications into unicompartmental knee, total knee, and total hip replacement. The first case of a revision surgery from conventional unicompartmental to total knee arthroplasty with the utilization of the robotic arm-assisted MAKO system is presented. An 87-year-old female presented with deteriorating left knee pain due to failure of medial unicompartmental knee arthroplasty at the outpatient clinic. The patient was advised to undergo revision surgery. Through medial parapatellar arthrotomy, the joint was exposed. With the use of the MAKO system, the estimated depth of the medial plateau according to CT planning was found to be 10 mm more distal than the lateral. The resection line of the remaining plateau was placed deliberately 2 mm more distal in order to achieve satisfactory replacement of the bony gap of the medial tibial condyle by a 10 mm augment. The patient had an uneventful recovery. A plethora of additional applications in the future, such as total shoulder or reverse total shoulder arthroplasty, megaprosthesis placement in oncological patients, and total hip or knee revision surgeries, may improve patient-related outcomes.

## 1. Introduction

Robotic systems were introduced in surgery during the early 1990s [1]. Robotic systems for orthopaedic surgery were developed in 1992, initially for assistance in total hip replacement [1, 2]. The expectation for better surgical outcomes proved to be at the beginning a great disappointment due to significant technical problems. Robotic systems are classified into two categories: autonomous and haptic (or surgeon-guided) [1, 3]. Passive surgery systems, which represent an alternative type of technology, are still questionable whether they are robotic or just computer-assisted [1, 4, 5].

Autonomous robotic systems were gradually withdrawn in the late 90s following the huge disappointment with the system ROBODOC [1]. However, the haptic tactile systems with technological improvements have gained wide acceptance. The most significant haptic tactile system is the Robotic Arm Interactive Orthopedic MAKO Stryker (RIO; MAKO Stryker, Fort Lauderdale, Florida) system, counting over 700 systems, in use, worldwide. The potential for improving surgical outcomes with the use of the robotic arm-assisted arthroplasty system has already been documented [1, 6–9].

The first application for robotic arm-assisted technology in 2006 was the partial knee arthroplasty, an operation where precision and preoperative planning may prove to be a real game changer. The next application was the total hip arthroplasty (THA) in 2008, and finally, in 2017, the total knee arthroplasty (TKA) completed the whole spectrum of knee and hip replacement surgery [1]. The MAKO robotic arm-assisted technology is the only FDA-approved technology for both knee and hip applications.

A case of an 87-year female undergoing revision surgery from manual medial unicompartmental knee arthroplasty to robotic-assisted total knee arthroplasty (RATKA) is presented. To the best of our knowledge, this is the first case reporting a knee arthroplasty revision utilizing the robotic arm-assisted system.

## 2. Case Presentation

An 87-year-old female was referred to the outpatient orthopaedic clinic, due to intense deteriorating left knee pain. The patient had undergone a medial manual unicompartmental left knee arthroplasty 1.5 years ago, due to medial osteoarthritis of the knee joint. Her body mass index was 25 kg/m^2^ (1.65/68), while the remaining medical history was unremarkable.

**Figure 1:**
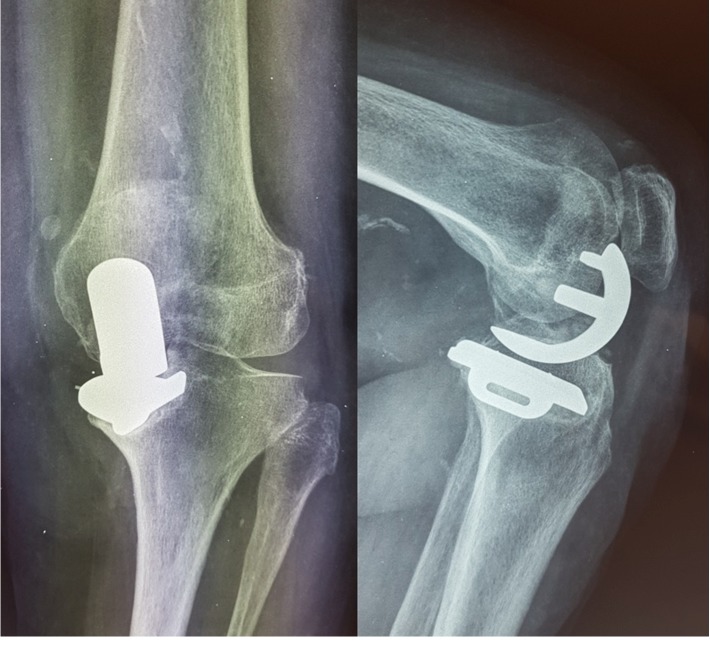
Anteroposterior and lateral X-ray views during initial evaluation. Mal-positioning of the unicompartmental knee arthroplasty with varus deformity is observed.

Physical examination revealed a painful restriction of range of motion, with a deficit of extension 10° and flexion restricted to 115°. Rotation movements were normal, but there was significant medial instability due to the 9° varus deformity. Patellofemoral joint motion and patellar tracking were found normal. Anteroposterior and lateral radiographs of the left knee confirmed the varus deformity, the suboptimal position of the prosthesis, the deteriorated tracking between the femoral and tibial components, and the elevation of the medial part of the tibial implant. These factors had contributed significantly to the early failure of the partial knee arthroplasty (Figure 1).

The patient was counseled on treatment options, including surgical management and elected to undergo revision arthroplasty surgery with the robotic arm-assisted system. Preoperative computer tomography (CT) scan for the robotic arm-assisted arthroplasty procedure and for further evaluation was performed. CT scanning was used to perform preoperative implant planning using patient-specific CT-based bone model and virtual implant templates.

Under epidural anesthesia, the patient was placed in the supine position. The leg was sterilized, draped with the usual instrumentation as in every primary RATKA, and secured using a leg positioner. Tourniquet was applied but not inflated. By extending the patient's previous incision, an anterior approach to the left knee was performed. Then, through medial parapatellar arthrotomy, the joint was exposed. Anterior cruciate ligament, Hoffa's fat pad, and lateral meniscus were resected. Posterior cruciate ligament was preserved. At that point, placement of the arrays took place as in primary RATKA. Bone registration, including the patient's landmarks, bone checkpoints, and verification, was conducted. The cortical bone's segmentation was similar to any primary RATKA with the assumption that femoral and tibial components were the native femoral and tibial condyles and cartilage.

According to preoperative planning, distal femur medial resection was 8.0 mm and lateral 2.0 mm. Posterior femur medial resection was 10.5 mm medial and lateral resection was 9.5 mm. Posterior Condylar Axis (PCA) was 1.8°, while the transepicondylar axis (TEA) was 0°. The system showed that the cuts' depth of the lateral compartment of the tibial plateau should be 7 mm.

**Figure 2:**
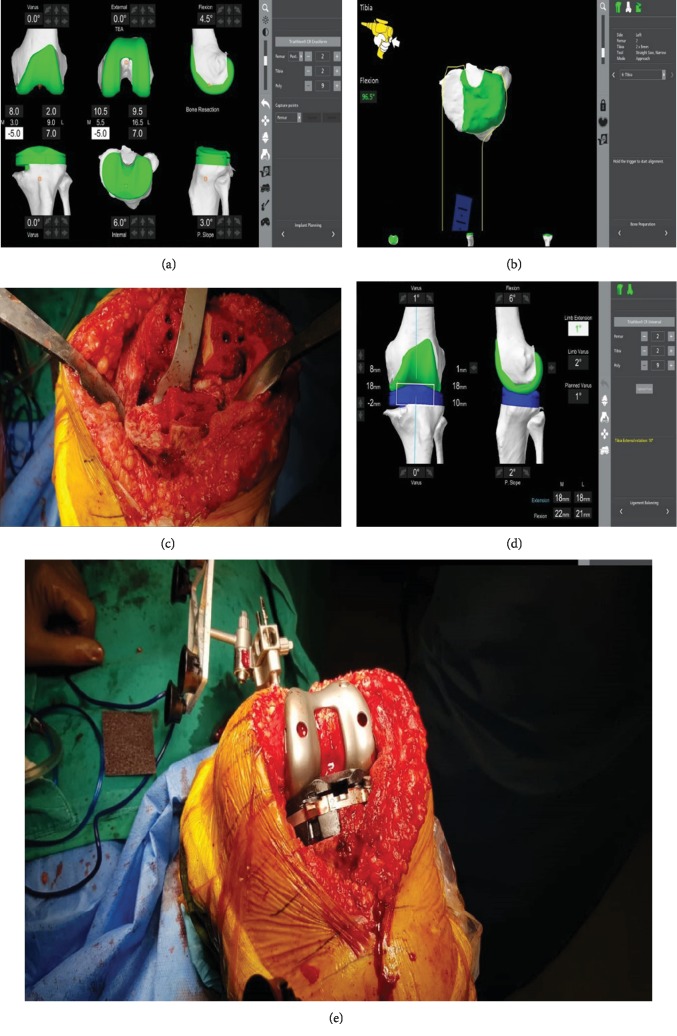
(a) Preoperative planning: distal femur medial resection was 8.0 mm and lateral 2.0 mm. Posterior femur medial resection was 10.5 mm medial and lateral resection was 9.5 mm. Posterior Condylar Axis (PCA) was 1.8°. Transepicondylar axis (TEA) was 0°. (b) Tibial cut was performed with the consideration of moving the resection line of the tibial cut 2 mm distally. (c) Intraoperative picture at that point. (d) Control of stability and range of motion was performed. (e) Final intraoperative picture following prosthesis placement.

Prior to prosthesis removal, balancing and measurements' evaluation were performed. Following the surgeon's corrections, the estimated depth of the lateral tibial plateau depth was 10 mm. Additionally, the femoral's medial compartment from 10 mm was corrected to 9.5, TEA was 1°, flexion from 4.5° became 6°, and the slope was from 3° to 2°. The estimated depth of the medial plateau according to CT planning was found to be 10 mm more distal than the lateral. The resection line of the remaining plateau was placed deliberately 2 mm more distal to achieve satisfactory replacement of the bony gap of the medial tibial condyle by the 10 mm augment (Figure 2).

The tibial component was removed first with the use of fine osteotomes in order to preserve as much bone as possible. Subsequently, the femoral component was removed using a Gigli saw and fine osteotomes. The remaining bone cuts on both the femur and tibia were performed under the guidance of the robotic arm-assisted system. The femoral cut was performed first. Special attention was paid in order to avoid notching of the anterior femoral cortex. The tibial cut was performed with special consideration of the modified operative plan (the resection line of the tibial cut was moved 2 mm distally than anticipated in primary RATKA in order to fill the gap of the medial condyle with the maximum provided 10 mm augment). The freehand fine-cut osteotomy was performed in order to assure the augment's correct placement.

Consequently, trials were placed on both the femur and tibia, and control of stability and range of motion were performed and registered by the robotic arm-assisted arthroplasty system. The 11 mm polyethylene instead of the 9 mm was used, in order to compensate the extra 2 mm of bone resection and to restore the joint line.

At this point, a sterilized rubber band was used and the tourniquet was inflated. Thereafter, trials were removed and replaced by the final cemented components. Femoral and tibial final implants were Triathlon Stryker No. 2 (cemented). Additional tests of stability and range of motion were performed in order to verify satisfactory soft tissue balancing. The wound was closed in layers in the usual fashion.

**Figure 3:**
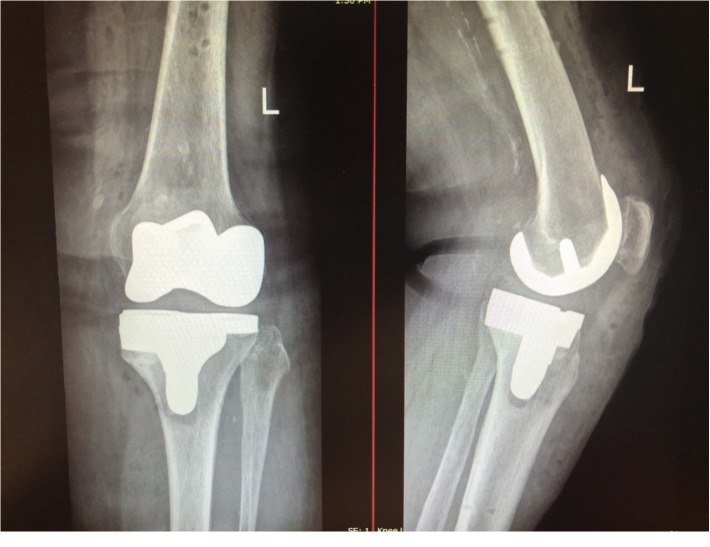
Postoperative anteroposterior and lateral X-ray views.

The patient made an uneventful recovery and was discharged at the second postoperative day. She was commenced on physiotherapy from the 1^st^ postoperative day, with a continuous passive motion device, as well as active kinesiotherapy. Six months postoperatively, the patient had a normal and painless pattern of gait together with full extension and flexion of 130° (Figure 3). At the last follow-up (1 year postoperation), she is very satisfied. Walking without a walking aid is possible. Flexion of the knee is 130° and the patient can climb stairs.

## 3. Discussion

Robotic arm-assisted arthroplasty was introduced in Orthopaedics in 2006, representing a haptic tactile system and since then has expanded its applications into unicompartmental knee (UKA), total knee, and hip arthroplasty [1]. Regarding UKA, robotic arm-assisted procedures have shown to overcome technical challenges associated with manual partial knee procedures, increasing accuracy in recreating the posterior tibial slope and coronal tibial alignment and, therefore, leading to better midterm clinical outcomes [6, 7, 10–12]. Regarding THA, studies have shown that robotic arm-assisted THA improved accuracy for both acetabular abduction angles, as well as acetabular anteversion, and demonstrated lower dislocation rates, when compared to manual THA [12–15]. Finally, RATKA has shown very promising short-term results [8]. In this present paper, we described the first revision from UKA to TKA with the off-label utilization of the robotic arm-assisted arthroplasty platform enhanced with a single-stage manual intervention.

Although knee revision with the robotic arm-assisted arthroplasty system is not licensed and not approved, we were tempted to benefit from the accuracy of this system for the preoperative planning and execution of the bone cutting. It is of note that we used the existing software for primary RATKA. However, the resection line of the tibial cut was moved 2 mm distally during intraoperative planning than anticipated in the primary RATKA in order to fill the gap of the medial condyle with the 10 mm augment. The new virtual cutting line of the medial tibia was manually optimized in order to reach the healthy intact part of the bone for the augment. It is of note that the robotic arm-assisted arthroplasty system allows the surgeon to change or adjust the implant placement and size, intraoperatively, at any stage. In the present case, due to lack of software for knee revisions, manual intervention was necessary for the medial tibial cut.

Robotic arm-assisted cutting and reaming may have many more applications in the future, such as total shoulder or reverse total shoulder arthroplasty, megaprosthesis placement in oncological patients, and total hip or knee revision surgeries. Improvements, especially in the system's software towards this direction, are of paramount importance. The present case represents, to our knowledge, the first successful revision of a manual unicompartmental knee to total knee arthroplasty with the utilization of the robotic arm-assisted system, expanding, in a way, the system's possible applications. Nevertheless, the robotic arm-assisted arthroplasty system should firstly be approved and licensed for joint knee and hip revision surgeries in order to be used systematically in such procedures.
